# Tauroursodeoxycholic Acid Protects against the Effects of *P*-Cresol-Induced Reactive Oxygen Species via the Expression of Cellular Prion Protein

**DOI:** 10.3390/ijms19020352

**Published:** 2018-01-25

**Authors:** Seung Pil Yun, Yeo Min Yoon, Jun Hee Lee, Minjee Kook, Yong-Seok Han, Seo Kyung Jung, Sang Hun Lee

**Affiliations:** 1 Department of Neurology, Institute for Cell Engineering, Johns Hopkins University School of Medicine, Baltimore, MD 21205, USA; spyun1002@gmail.com (S.P.Y.); mkook2@jhu.edu (M.K.); 2 Institute of Soonchunhyang Medical Science Research, Soonchunhyang University Hospital Seoul, Soonchunhyang University, Seoul 04401, Korea; yoonboo15@naver.com (Y.M.Y.); format7000@naver.com (Y.-S.H.); 3569123@naver.com (S.K.J.); 3 Department of Pharmacology and Toxicology, University of Alabama at Birmingham School of Medicine, Birmingham, AL 35294, USA; j-school@hanmail.net

**Keywords:** mesenchymal stem cells, *P*-cresol, tauroursodeoxycholic acid, cellular prion protein, reactive oxygen species

## Abstract

Mesenchymal stem cells (MSCs) could be a promising solution in the treatment of various diseases including chronic kidney disease (CKD). However, endoplasmic reticulum (ER) stress induced by ischemia in the area of application limits the integration and survival of MSCs in patients. In our study, we generated ER stress-induced conditions in MSCs using *P*-cresol. As *P*-cresol is a toxic compound accumulated in the body of CKD patients and induces apoptosis and inflammation through reactive oxygen species (ROS), we observed ER stress-induced MSC apoptosis activated by oxidative stress, which in turn resulted from ROS generation. To overcome stress-induced apoptosis, we investigated the protective effects of tauroursodeoxycholic acid (TUDCA), a bile acid, on ER stress in MSCs. In ER stress, TUDCA treatment of MSCs reduced ER stress-associated protein activation, including GRP78, PERK, eIF2α, ATF4, IRE1α, and CHOP. Next, to explore the protective mechanism adopted by TUDCA, TUDCA-mediated cellular prion protein (PrP^C^) activation was assessed. We confirmed that PrP^C^ expression significantly increased ROS, which was eliminated by superoxide dismutase and catalase in MSCs. These findings suggest that TUDCA protects from inflammation and apoptosis in ER stress via PrP^C^ expression. Our study demonstrates that TUDCA protects MSCs against inflammation and apoptosis in ER stress by PrP^C^ expression in response to *P*-cresol exposure.

## 1. Introduction

Chronic kidney disease (CKD), as the name suggests, is caused by prolonged damage to the kidney, and patients with CKD suffer from progressive loss of kidney functions. An unhealthy kidney fails to metabolize, detoxify, and reabsorb organic waste solutes, and leads to the accumulation of uremic toxins in blood vessels. The accumulated toxins are transported to other organs and cause angiosis, anemia, pericarditis, neurologic ossification, or other neurological problems [1,2,3,4,5]. There are more than 100 uremic toxins that can be divided into two major categories. The first category represents the combination of water-soluble substances and serum proteins that are excreted from the body owing to the loss of resorption. The second category includes protein-bound solutes that can neither be removed from the body nor broken down; these solutes stay in the body for a long period [6,7]. *P*-cresol (PC) is an example of protein-bound uremic toxins, which cause cell senescence, apoptosis, and immunodeficiency in the cardiovascular system, brain, kidneys, and other organs of the affected patients. However, the effects of PC on adult stem cells in patients with CKD are not yet fully discovered.

Mesenchymal stem cells (MSCs) have been studied and regarded widely as a potential candidate for various cell-based therapies because of its differentiation ability, multipotency, and immunomodulatory activity [8,9]. However, the use of MSCs in cell-based therapy demands resistance to harsh conditions, such as oxidative stress at the treatment site, and immune response to the newly implanted cells [8,10]. Foreign substances including newly treated MSCs induce oxidative stress around the area of application, promote generation of reactive oxidative species (ROS) in physio-pathological conditions, and ultimately result in the endoplasmic reticulum (ER) stress that leads to apoptosis [11,12]. ER is an essential organelle responsible for homeostatic control, cellular survival, and apoptosis. When homeostasis in the ER cannot be maintained, cell death occurs, leading to the accumulation of immature proteins [13,14]. Specifically, IRE1α, a significant signaling pathway in ER stress, activates NF-κB, JNK, and p38 to induce inflammation in the cell, and ultimately promotes apoptosis [15,16]. In addition, the IRE1α signaling pathway is known to induce the PERK signaling pathway to promote the expression of CHOP, which causes apoptosis and decreases the effectiveness of cell-based therapy [17,18,19].

Cellular prion protein (PrP^C^) is a glycoprotein related to PrP^SC^, which is a misfolded isoform proteinase resistant protein and causes a neurodegenerative disease called prion [20,21]. PrP^C^ plays an important role in the survival of MSCs by reducing the oxidative stresses in an ischemia model [22]. Supporting studies suggest that it is necessary for neurogenesis, differentiation, and proliferation of a cell [23], controlling expression of proteins related to extracellular matrix in order to increase the adhesiveness of a cell [24], and resisting the oxidative stress by combining with Cu^2+^ and suppressing the formation of ROS [22,25]. Therefore, the increase in PrP^C^ expression is an interesting target, which could be utilized to promote the survival of stem cells exposed to PC in oxidative stress.

Tauroursodeoxycholic acid (TUDCA) is a bile acid and a drug approved by the US Food and Drug Administration, and is thought to be effective in increasing the cell proliferation and treating neurodegenerative diseases, coxitis, angiosis, and diabetes [22,26,27,28,29]. Recent studies suggest that TUDCA can be used to maintain the homeostatic level of ER stress in the cell. TUDCA also resists imperfect differentiation and inflammation of stem cells caused by the extracellular change [29,30]. However, it is unknown whether TUDCA provides a defensive mechanism to stem cells against PC.

Previous studies have verified the potential of TUDCA in reducing the amount of ROS and ER stress in MSCs exposed to PC, in addition to the therapeutic effects of PrP^C^ in reducing inflammatory responses and apoptosis. These studies prompted us to investigate whether TUDCA protected against the effects *P*-Cresol-induced ROS via the expression of cellular prion protein. Our results demonstrated that TUDCA could potentially target uremic toxins generated by PC.

## 2. Results

### 2.1. TUDCA Inhibits PC-Induced ER Stress by Suppressing ROS Generation

**Figure 1:**
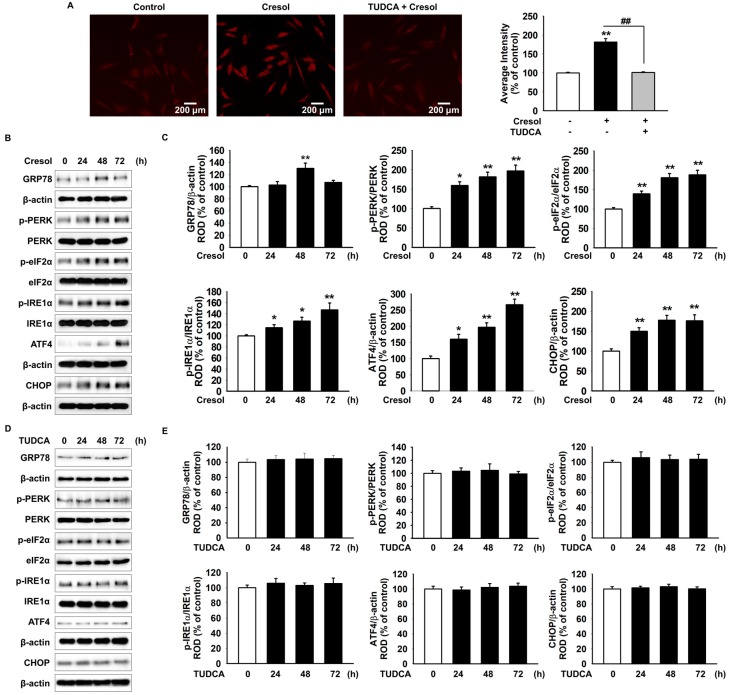

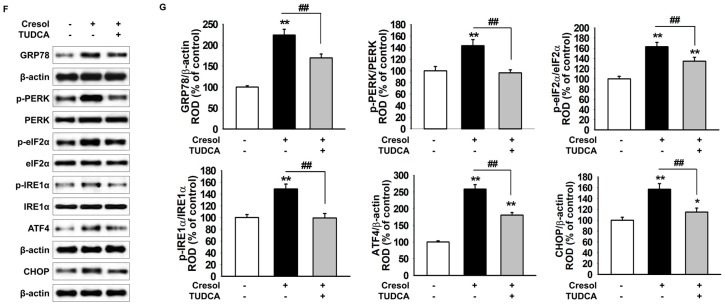
Protective effect of TUDCA on *P*-cresol-induced ER stress by suppressing the generation of reactive oxygen species. (**A**) Mesenchymal stem cell (MSCs) were treated with *P*-cresol (PC; 500 µM) for 72 h after they were pretreated with 100 µM TUDCA for 24 h. ROS levels after the 72-h PC exposure are represented by DHE fluorescence staining in different treatment groups. The right panel represents the right panel represents the percentage of average fluorescence intensity (*n* = 5). +: chemical treatment of MSCs, and −: no chemical treatment of MSCs. Values represent the mean ± SEM. ** *p* < 0.01 vs. untreated MSCs, ## *p* < 0.01 vs. H_2_O_2_ + MSCs. (ANOVA, using Tukey’s post-hoc test). (**B**) Western blot analysis detected GRP78, p-PERK, PERK, p-eIF2α, eIF2α, p-IRE1α, IRE1α, ATF4, and CHOP expression after the treatment of MSCs with PC for the indicated duration. (**C**) The expression of the target proteins was normalized to that of β-actin, PERK, eIF2α, and IRE1α (*n* = 3). Values represent the mean ± SEM. * *p* < 0.05 and ** *p* < 0.01 vs. untreated MSCs. (ANOVA, using Dunnett’s post-hoc test). (**D**) Western blot shows GRP78, p-PERK, PERK, p-eIF2α, eIF2α, p-IRE1α, IRE1α, ATF4, and CHOP expression after the treatment of MSCs with TUDCA (100 µM; 0, 24, 48, and 72 h). (**E**) Protein expression was normalized to that of β-actin, PERK, eIF2α, and IRE1α (*n* = 3). (ANOVA, using Dunnett’s post-hoc test). (**F**) Western blot shows GRP78, p-PERK, PERK, p-eIF2α, eIF2α, p-IRE1α, IRE1α, ATF4, and CHOP expression after the treatment of TUDCA-pretreated MSCs with PC for 72 h. +: chemical treatment of MSCs, and –: no chemical treatment of MSCs. (**G**) Protein expression was normalized to that of β-actin, PERK, eIF2α, and IRE1α (*n* = 3). +: chemical treatment of MSCs, and −: no chemical treatment of MSCs. Values represent the mean ± SEM. * *p* < 0.05 and ** *p* < 0.01 vs. untreated MSCs, ## *p* < 0.01 vs. treatment of MSCs with *P*-cresol. (ANOVA, using Tukey’s post-hoc test).

PC induces ER stress through the generation of ROS. To confirm whether TUDCA could suppress the PC-induced ROS generation, we treated MSCs with PC (500 µM) for 72 h in the presence or absence of TUDCA (100 µM) for 24 h. We measured ROS levels in the cells by dihydroethidium (DHE) staining, which involves a widely used specific fluorescent probe molecule for O_2_^−^. Our results demonstrated that TUDCA reduced ROS generation after PC exposure (Figure 1A). Next, as ROS generated by PC induced ER stress, we found that TUDCA reduced ER stress-associated proteins (78 kDa glucose-regulated protein (GRP78), protein kinase R-like endoplasmic reticulum kinase (PERK), eukaryotic initiation factor 2-alpha (eIF2α), activating transcription factor 4 (ATF4), inositol-requiring protein 1 alpha (IRE1 α), and CCAAT-enhancer-binding protein homologous protein (CHOP)) in MSCs exposed to PC. Exposure of MSCs with PC (500 µM) for different periods of time (0, 24, 48, and 72 h) enhanced the expression of ER stress markers (GRP78, ATF4, and CHOP) and regulators (p-PERK, p-eIF2α, and p-IRE1α) (Figure 1B,C). However, TUDCA alone did not affect the expression of ER stress markers in MSCs (Figure 1D,E). To verify whether TUDCA prevented ER stress in PC-induced ROS, we estimated ER stress-associated proteins in MSCs after the 72- treatment with PC (500 µM) in the presence or absence of TUDCA (100 µM) for 24 h by western blotting. MSCs pretreated with TUDCA had significantly inhibited ER stress-associated proteins (Figure 1F,G). These results suggest that TUDCA inhibits the activation of ER stress by PC-induced ROS.

### 2.2. TUDCA Inhibits PC-Induced ROS Generation *via* Cellular Prion Protein-Dependent Catalase and SOD Activation

**Figure 2:**
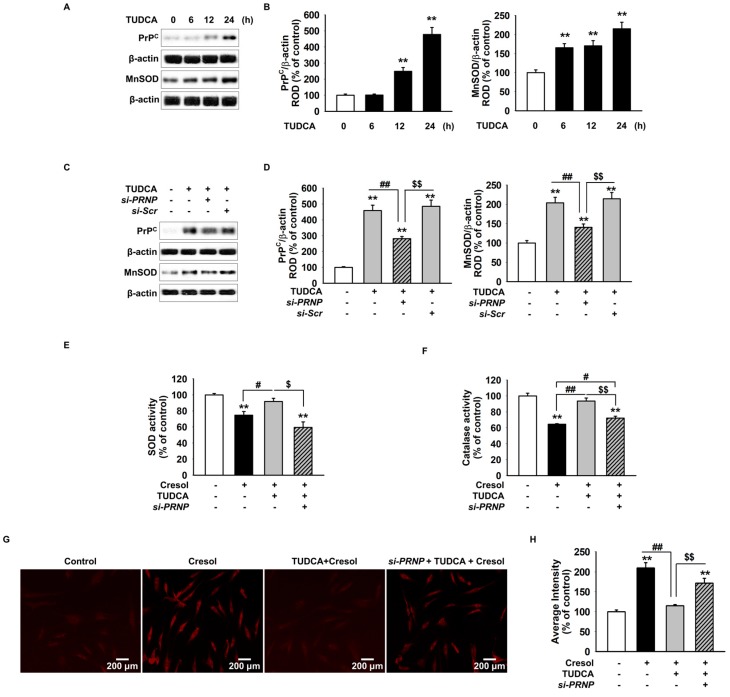
Protective effect of TUDCA on P-cresol-induced generation of reactive oxygen species via the expression of PrP^C^. (**A**) MSCs were treated with TUDCA (100 µM) at regular intervals (0, 6, 12, and 24 h). Western blot detected PrP^C^ and MnSOD expression after the treatment of MSCs with TUDCA for the indicated duration. (**B**) The expression of target proteins was normalized to that of β-actin (*n* = 3). Values represent the mean ± SEM. ** *p* < 0.01 vs. untreated MSCs. (ANOVA, using Dunnett’s post-hoc test). (**C**) Western blot shows PrP^C^ and MnSOD expression after the treatment of *si-PRNP*-transfected MSCs with TUDCA for 24 h. +: chemical treatment of MSCs, and −: no chemical treatment of MSCs. (**D**) Protein expression was normalized to that of β-actin (*n* = 3). Values represent the mean ± SEM. +: chemical treatment of MSCs, and −: no chemical treatment of MSCs. ** *p* < 0.01 vs. untreated MSCs, ## *p* < 0.01 vs. treatment of MSCs with TUDCA, $$ *p* < 0.01 vs. TUDCA-treated MSCs transfected with *si-PRNP*. (ANOVA, using Tukey’s post-hoc test). (**E**,**F**) After exposure with *P*-cresol, ROS levels, catalase activity, and SOD activity were measured in MSCs treated with TUDCA by determining the expression level of PrP^C^ (*n* = 3). +: chemical treatment of MSCs, and −: no chemical treatment of MSCs. Values represent the mean ± SEM. ** *p* < 0.01 vs. untreated MSCs, # *p* < 0.05 and ## *p* < 0.01 vs. treatment of MSCs with *P*-cresol, $ *p* < 0.05 and $$ *p* < 0.01 vs. treatment of MSCs with TUDCA. (ANOVA, using Tukey’s post-hoc test). (**G**) After *P*-cresol exposure for 72 h, ROS levels were analyzed by DHE staining in *si-PRNP*-transfected MSCs after the treatment with TUDCA for 24 h. (**H**) The right panel represents the number of DHE-positive cells per high-power field (*n* = 5). +: chemical treatment of MSCs, and −: no chemical treatment of MSCs. Values represent the mean ± SEM. ** *p* < 0.01 vs. untreated MSCs, ## *p* < 0.01 vs. treatment of MSCs with *P*-cresol, $$ *p* < 0.01 vs. treatment of MSCs with TUDCA. (ANOVA, using Tukey’s post-hoc test).

Our findings were consistent with the previous results that treatment of MSCs with TUDCA increases the expression of PrP^C^ via the Akt signaling pathway. To confirm whether TUDCA directly reduced the PC-induced ROS generation, we measured the expression of PrP^C^ and MnSOD in MSCs after the treatment with TUDCA at regular intervals (0, 6, 12, and 24 h) by Western blotting. We found that the expression of PrP^C^ and MnSOD increased in a time-dependent manner (Figure 2A,B). To confirm whether the increased PrP^C^ levels by TUDCA regulated the expression of MnSOD, we blocked the expression of PrP^C^ using *si-PRNP*. Expression of MnSOD decreased in MSCs transfected with *si-PRNP* despite the presence of TUDCA (Figure 2C,D). Furthermore, treatment of MSCs with PC increased SOD and catalase activation in the presence of TUDCA compared with that in the absence TUDCA. As decreased SOD and catalase activity by blocking expression of PrP^C^ established this effect was PrP^C^ regulation of ROS activity by treatment with TUDCA (Figure 2E,F). DHE staining revealed an increased accumulation of ROS in MSCs pretreated with *si-PRNP*. (Figure 2G,H). These results suggested that TUDCA reduced ROS generation through increased SOD and catalase activity via the expression of PrP^C^.

### 2.3. TUDCA Exerts Protective Effect on PC-Induced Apoptosis of MSCs

**Figure 3:**
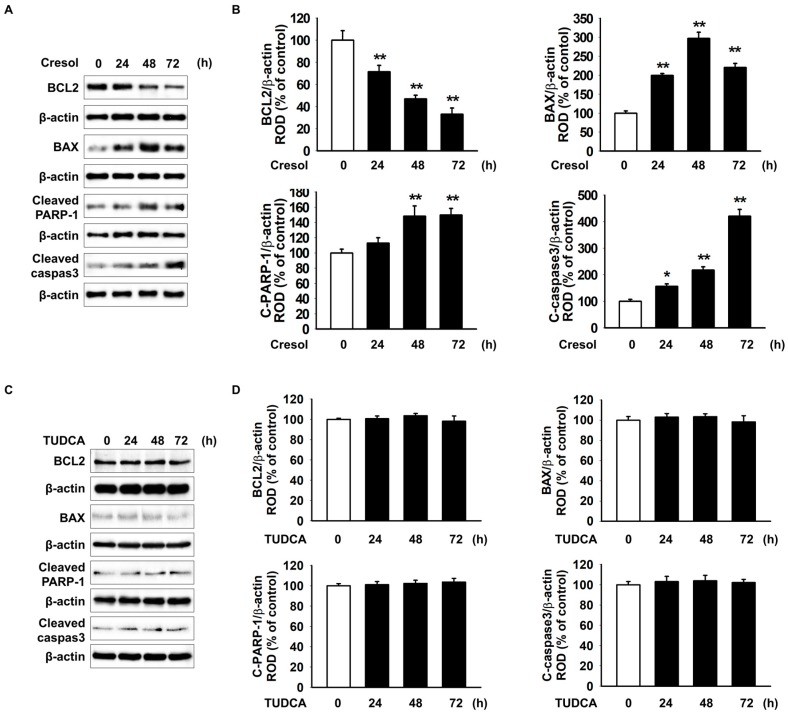

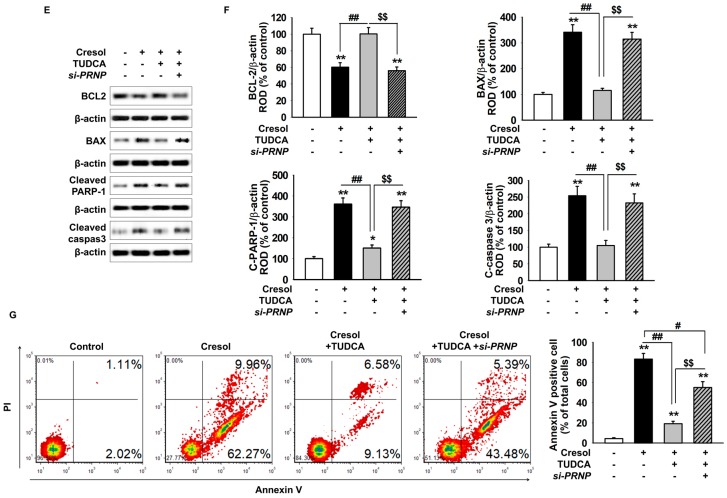
Protective effect of TUDCA on *P*-cresol-induced apoptosis in ER stress via the expression of PrP^C^. (**A**) Western blot shows BCL2, BAX, cleaved PARP-1, and cleaved caspase-3 expression after the treatment of MSCs with *P*-cresol for the indicated duration. (**B**) Protein expression was normalized to that of β-actin (*n* = 3). Values represent the mean ± SEM. * *p* < 0.05 and ** *p* < 0.01 vs. untreated MSCs. (ANOVA, using Dunnett’s post-hoc test). (**C**) Western blot shows BCL2, BAX, cleaved PARP-1, and cleaved caspase-3 expression after the treatment of MSCs with TUDCA (100 µM; 0, 24, 48, and 72 h). (**D**) Protein expression was normalized to that of β-actin (*n* = 3). (ANOVA, using Dunnett’s post-hoc test). (**E**) After the 72-h *P*-cresol exposure, Western blot shows BCL2, BAX, cleaved PARP-1, and cleaved caspase-3 expression in MSCs after the 24-h treatment of *si-PRNP*-transfected MSCs with TUDCA. +: chemical treatment of MSCs, and −: no chemical treatment of MSCs. (**F**) Protein expression was normalized to that of β-actin (*n* = 3). +: chemical treatment of MSCs, and −: no chemical treatment of MSCs. Values represent the mean ± SEM. * *p* < 0.05 and ** *p* < 0.01 vs. untreated MSCs, ## *p* < 0.01 vs. treatment of MSCs with *P*-cresol, $$ *p* < 0.01 vs. treatment of MSCs with TUDCA. (ANOVA, using Tukey’s post-hoc test). (**G**) Apoptosis in MSCs was analyzed by Annexin V/PI staining and flow cytometer-based analysis. +: chemical treatment of MSCs, and −: no chemical treatment of MSCs. (*n* = 5). Values represent the mean ± SEM. ** *p* < 0.01 vs. untreated MSCs, # *p* < 0.05 and ## *p* < 0.01 vs. treatment of MSCs with *P*-cresol, $$ *p* < 0.01 vs. treatment of MSCs with TUDCA. (ANOVA, using Tukey’s post-hoc test).

PC decreased the expression of the anti-apoptotic protein BCL-2 and increased the expression of the pro-apoptotic protein BAX, cleaved PARP-1, and cleaved caspase-3 in a time-dependent manner (Figure 3A,B). However, TUDCA alone did not affect the expression of BCL2, BAX, C-PARP-1 and C-caspase3 in MSCs (Figure 3C,D). In addition, MSC treatment with TUDCA resulted in an increase in the expression of BCL-2 and a significant decrease in expression of BAX, PARP-1 and cleaved caspase-3 expressed by *P*-cresol. The protective effect on MSCs was also sensitive to the blocked expression of PrP^C^ (Figure 3E,F). We analyzed apoptotic cells using the Annexin V/PI assay and found that apoptotic MSCs decreased after the treatment with TUDCA compared with that in other groups after PC exposure (Figure 3G). These results indicated that TUDCA prevented PC-induced ROS and apoptosis by increasing the expression of PrP^C^.

### 2.4. TUDCA Prevents PC-Induced Inflammation via the Expression of PrP^C^

**Figure 4:**
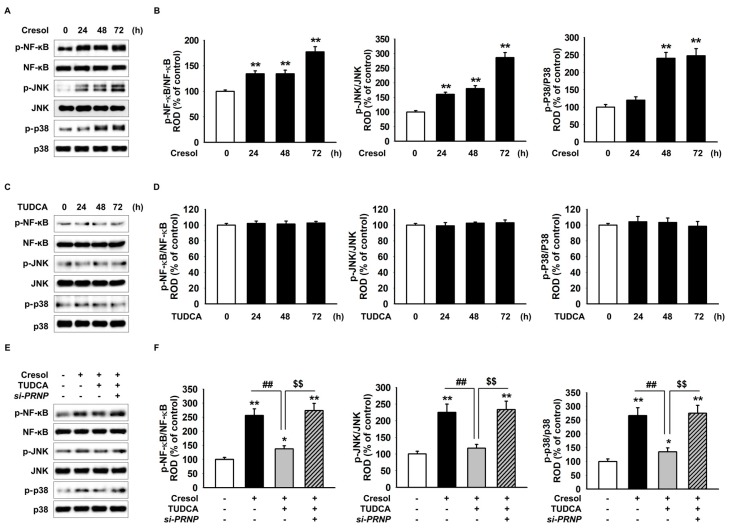
Protective effect of TUDCA on *P*-cresol-activated inflammation pathway through ER stress via the expression of PrP^C^. (**A**) Western blot shows p-NF-κB, NF-κB, p-JNK, JNK, p-p38, and p38 expression after the treatment of MSCs with *P*-cresol for the indicated duration. (**B**) Protein expression was normalized to that of NF-κB, JNK, and p38 (*n* = 3). Values represent the mean ± SEM. ** *p* < 0.01 vs. untreated MSCs. (ANOVA, using Dunnett’s post-hoc test). (**C**) Western blot shows p-NF-κB, NF-κB, p-JNK, JNK, p-p38 and p38 expression after the treatment of MSCs with TUDCA (100 µM; 0, 24, 48, and 72 h). (**D**) Protein expression was normalized to that of β-actin, NF-κB, JNK, and p38 (*n* = 3). (ANOVA, using Dunnett’s post-hoc test). (**E**) After *P*-cresol exposure for 72 h, Western blot shows p-NF-κB, NF-κB, p-JNK, JNK, p-p38, p38 expression after the 24-h treatment of *si-PRNP*-transfected MSCs with TUDCA. +: chemical treatment of MSCs, and −: no chemical treatment of MSCs. (**F**) Protein expression was normalized to that of NF-κB, JNK, and p38 (*n* = 3). +: chemical treatment of MSCs, and −: no chemical treatment of MSCs. Values represent the mean ± SEM. * *p* < 0.05 and ** *p* < 0.01 vs. untreated MSCs, ## *p* < 0.01 vs. treatment of MSCs with *P*-cresol, $$ *p* < 0.01 vs. treatment of MSCs with TUDCA. (ANOVA, using Tukey’s post-hoc test).

**Figure 5:**
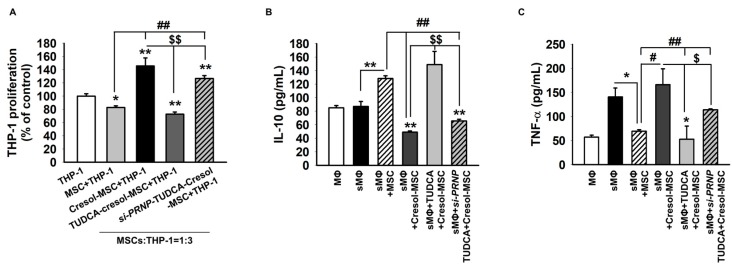
TUDCA attenuated pro-inflammatory cytokine via the expression of PrP^C^. (**A**) THP-1 cells were cultured alone (THP-1) or with MSCs (MSC + THP-1), MSCs exposed to *P*-cresol (Cresol-MSCs + THP-1), TUDCA-treated MSCs exposed to *P*-cresol (TUDCA-MSCs + THP-1), or *si-PRNP*-transfected MSCs with TUDCA and exposed to *P*-cresol (*si-PRNP*-TUDCA-MSCs + THP-1) using culture inserts, after which proliferation of THP-1 cells was assessed (*n* = 3). Values represent the mean ± SEM. * *p* < 0.05 and ** *p* < 0.01 vs. THP-1, ## *p* < 0.01 vs. MSC + THP-1, $$ *p* < 0.01 vs. TUDCA-Cresol-MSCs + THP-1. (ANOVA, using Tukey’s post-hoc test). (**B**,**C**) Macrophages (ΜΦ) were cultured alone, and LPS-stimulated macrophages were either cultured alone (sΜΦ) or co-cultured with MSCs (sΜΦ + MSC), MSCs exposed to *P*-cresol (sΜΦ + Cresol-MSCs), TUDCA-treated MSCs exposed to *P*-cresol (sΜΦ + TUDCA-Cresol-MSCs), or *si-PRNP*-transfected MSCs with TUDCA and exposed to *P*-cresol (sΜΦ + *si-PRNP*-TUDCA-Cresol-MSCs) for 48 h, after which IL-10 and TNF-α levels were determined by ELISA (*n* = 3). Values represent the mean ± SEM. * *p* < 0.05 and ** *p* < 0.01 vs. sΜΦ, # *p* < 0.05 and ## *p* < 0.01 vs. sΜΦ + MSC, $ *p* < 0.05 and $$ *p* < 0.01 vs. ΜΦ + TUDCA-Cresol-MSCs. (ANOVA, using Tukey’s post-hoc test).

The IRE1α pathway is associated with ER stress and stimulates inflammation-associated proteins, NF-κB, JNK, and p38. These results confirm that ROS generation activates IRE1α by PC. Therefore, we predict that TUDCA exerts its protective effect against inflammation in PC-treated MSCs via the expression of PrP^C^. Inflammation activation proteins, such as p-NF-κB, p-JNK, and p-p38, increased after PC exposure in a time-dependent manner (Figure 4A,B). In contrast, TUDCA alone did not affect the expression of p-NF-κB, p-JNK, and p-p38 in MSCs (Figure 4C,D). However, to elucidate the protective effect of TUDCA after PC treatment, Western blotting was carried out. It showed that p-NF-κB, p-JNK, and p-p38 decreased in the presence of TUDCA compared to those in the absence of TUDCA (Figure 4E,F). As blocked expression of PrP^C^ neutralized the effects of TUDCA, it exerted its protective effect on PC via PrP^C^ expression (Figure 4E,F). Finally, we confirmed the effect of TUDCA on proliferation of macrophages in co-culture with MSCs using a culture insert dish. Proliferation of macrophages was attenuated in the co-culture of THP-1 with TUDCA-treated MSCs compared with that in both the co-culture of THP-1 with MSC and the co-culture of THP-1 with *si-PRNP*-transfected MSCs with TUDCA after PC exposure (Figure 5A). In addition, to investigate whether TUDCA regulates anti-inflammatory and pro-inflammatory cytokines in co-cultures of THP-1 and MSCs via PrP^C^, we analyzed the expression of TNF-α and IL-10 in macrophages by ELISA. Co-culture of THP-1 with TUDCA-treated MSCs significantly suppressed the expression of TNF-α and increased the expression of IL-10 compared with those in other co-culture treatment groups after PC exposure (Figure 5B,C). These results indicated TUDCA prevented inflammation resulting from PC-induced ROS by regulated expression of PrP^C^.

## 3. Discussion

Previous studies have suggested potential application of MSCs in the treatment of injured tissues [31,32]. However, there are limitations of such applications because of poor conditions near the application area from pathophysiological conditions including high ROS levels, inflammation, and apoptosis [33]. Our study thus aimed to investigate the potential of TUDCA for preventing apoptosis due to ROS in MSCs. Our results suggest that PC generates ROS in the cell and induces ER stress leading to immune responses and apoptosis. Furthermore, we confirmed that preprocessed TUDCA had the ability to prevent PC-induced and ROS-mediated damage by increasing the expression of PrP^C^.

PC is normally excreted from the body as a uremic toxin. However, it fails to be metabolized and accumulates in the bodies of patients suffering from CKD [1]. Specifically, a previous study on intracellular endothelial cells elucidated that the accumulation of PC in the body generates ROS inside the cells, decreases the expression of endothelial adhesion substance [34,35], increases the permeability and immune response [35,36,37], and enhances ER stress, leading to apoptosis and necrosis [34,37]. Therefore, PC has been used in our study to induce similar deterrence in MSC growth in injured tissues.

Our results suggest that PC induces ROS generation, thereby inducing production of ER stress-related proteins—GRP78, PERK, eIF2α, ATF4, and CHOP—resulting in apoptosis of potential MSCs. ROS is widely known to play a key role in the determination of cellular survival or death [38,39]. In our study, we used DHE staining to demonstrate that *P*-cresol increased ROS levels in MSCs. Supporting the previous literature, DHE results in our study showed that the pretreatment with TUDCA did not allow such ROS accumulation [22] and that there was a significant difference in ROS levels in PC-treated MSCs and PC-treated MSCs with TUDCA (Figure 1A). Previous studies suggest that ROS specifically works through the ER stress pathway to induce apoptosis. An ER chaperon protein, GRP78, is previously known to play an important role in cell survival and death by interacting with PERK [40]. GRP78 is known to be normally bound to PERK. However, in a stressed state, PERK dissociates from GRP78 and further activates the eIF2α-ATF4 pathway that eventually leads to apoptosis [41]. Similarly, we showed through Western blot analysis that TUDCA reduced the amount of ER stress-related proteins including GRP78, PERK, eIF2α, ATF4, CHOP, which are increased upon increment in ROS levels.

PrP^C^ is known to have a positive effect on cells in ischemia and hypoxia, conditions similar to those with ROS. Deficiency of PrP^C^ has been associated with the sensitivity to oxidative stress and subsequent apoptosis because of an increased caspase-3 activity [42]. PrP^C^ is thought to be positively associated with the activation of Akt and negatively associated with the apoptotic responses due to caspase-3 [42]. PrP^C^-induced Akt-mediated MnSOD activation is further known to protect MSCs against oxidative stress both in vitro and in vivo [43]. Our results supported that the levels of PrP^C^ and MnSOD were increased in MSCs after the treatment with TUDCA in a dose-dependent manner. Further, PrP^C^ knockdown using *si-PRNP* demonstrated that TUDCA-induced activation of MnSOD and catalase was dependent on the PrP^C^ pathway and that PC-induced ROS levels were not decreased by TUDCA when PrP^C^ was knocked down. Therefore, we conclude that the protective effect of TUDCA is suppressed when PrP^C^ is knocked down. Such results show that PC indirectly increases ER stress through the enhanced production of ROS inside the cell and that PrP^C^ is essential for TUDCA to combat the PC-induced effects.

Activation of IRE1α, an ER stress sensor protein, triggers inflammatory response factors such as JNK and NF-κB, leading to the activation of a macrophage that kills the cells [15,16,44,45]. In agreement with previous literature, our results confirmed that the ER stress induced by the increase in *P*-cresol resulted in similar apoptotic and inflammatory responses via the PERK-eIF2α-ATF4 and IRE1α signaling pathway. Western blotting showed that inflammation activation proteins, including p-NF-κB, p-JNK, and p-p38 were increased on PC exposure in a time-dependent manner, and TUDCA successfully overcame theses effects. As TUDCA is known to exert its therapeutic effects through PrP^C^, knockdown of PrP^C^ negated the positive effects of TUDCA in reducing inflammation via the PERK-eIF2α-ATF4 and IRE1α signaling pathway. Macrophages are activated by inflammation and cytokines associated with ER stress. Similarly, inflammation-induced macrophage activation was detected in THP-1 cultures, and it was found that TUDCA-treated MSC cultures had less associated proteins compared to those in the untreated MSC cultures. ELISA was performed to detect specific cytokines such as TNF-α and IL-10 secreted by macrophages; these cytokines were ultimately attenuated by TUDCA treatment of MSCs. Our results demonstrate that TUDCA, a chemical chaperon known to resist the activation of ER stress [22,40], further suppresses apoptosis via inflammatory pathway and that its therapeutic effects are mediated via inflammatory pathway.

As it was verified that PC-induced ER stress leads to inflammatory responses, we demonstrated that TUDCA decreased inflammation and apoptosis due to ER stress. The PERK-eIF2α-ATF4 ER stress signal pathway then increases the expression level of CHOP [46,47]. Highly expressed CHOP decreases the expression level of BCL-2 and increases the expression level of BAX, ultimately inducing apoptosis [48]. Similarly, our results suggest that PC induces apoptosis via these apoptosis-related proteins, and TUDCA inhibits their expression, directly substantiating the eventual anti-apoptotic effects of the drug.

Overall, we determined that TUDCA decreased ROS levels to activate the defense mechanism of MSCs. We also found that TUDCA suppressed apoptosis of MSCs and inflammatory responses induced by ER stress, which was experimentally induced by *P*-Cresol. Using the knockdown of PrP^C^, we also demonstrated that PrP^C^ increase by TUDCA was an essential factor in the defense mechanism of PC. Therefore, we found that TUDCA-induced PrP^C^ helped MSCs resist the activation of ROS. Moreover, TUDCA decreased ER stress due to *P*-Cresol in CKD-related necrocytosis and inflammatory responses. The present study indicates that TUDCA has therapeutic potential to prevent secondary injuries caused by CKD. Further studies are required to examine these therapeutic effects of PrP^C^ on CKD.

## 4. Materials and Methods

### 4.1. MSC Culture

Human adipose tissue-derived MSCs were obtained from the American Type Culture Collection (Manassas, VA, USA), and were confirmed pathogen- and mycoplasma-free. MSCs expressed specific cell surface marker (CD73 and CD105), and showed adipogenic and osteogenic differentiation potential when cultured with specific differentiation media. MSCs were cultured in alph-minimum essential medium (Gibco BRL, Gaithersburg, MD, USA) supplemented with 10% (*v*/*v*) fetal bovine serum (FBS; Gibco BRL), 100 U/mL of penicillin, and 100 µg/mL of streptomycin. MSCs were grown in a humidified incubator with 5% CO_2_ at 37 °C.

### 4.2. Chemical Treatments

MSCs were washed twice with phosphate buffer saline (PBS), and fresh α-MEM supplemented with 10% FBS was added. To investigate the ER stress signaling pathway, MSCs were pretreated with TUDCA (100 µM) at 37 °C for 24 h and then treated with *P*-cresol (500 µM) at regular intervals (0, 24, 48 and 72 h).

### 4.3. Dihydroethidium (DHE) Staining

DHE (Sigma-Aldrich, St. Louis, MO, USA) was used to measure superoxide anion levels in cultured MSCs. The cells were incubated with DHE (10 µM) for 30 min. After washing with PBS twice, samples were visualized under a fluorescence microscope (Zeiss, Oberkochen, Germany) at 488 nm excitation and 590 nm emission.

### 4.4. Western Blot Assay

The MSCs were lysed using RIPA lysis buffer (Thermo Fisher Scientific, Waltham, MA, USA) to obtain total cellular protein. Cell lysates (20 µg of total protein) were separated on 8–12% sodium dodecyl sulfate polyacrylamide gel electrophoresis (SDS-PAGE), and the proteins were transferred to the nitrocellulose membrane. After the blots had been washed with TBST (10 mM Tris-HCl (pH 7.60), 150 mM NaCl, 0.05% Tween-20), they were blocked with 5% skimmed milk for 1 h and incubated with appropriate primary antibodies at the dilutions recommended by the supplier. Antibodies against GRP78, PERK, p-PERK, eIF2α, p-eIF2α, ATF4, IRE1α, CHOP, p-IRE1α, NF-κB, p-NF-κB, JNK, p-JNK, p38, p-p38, BCL-2, BAX, cleaved caspase-3, cleaved PARP-1, PrP^C^, MnSOD, and β-actin were purchased from Santa Cruz Biotechnology (Dallas, TX, USA). The membranes were then washed, and primary antibodies were detected using goat anti-rabbit IgG or goat anti-mouse IgG conjugated to horseradish peroxidase (Santa Cruz Biotechnology). The immunoreactive bands were visualized by enhanced chemiluminescence (Amersham Pharmacia Biotech, Little Chalfont, UK).

### 4.5. Inhibition of PrP^C^ Expression by RNA Interference

MSCs (2 × 10^5^ cells) were seeded in 60-mm dishes and transfected with *siRNA* in serum-free Opti-MEM (Gibco BRL) using Lipofectamine 2000 following the manufacturer’s instructions (Thermo Fisher Scientific). After 48 h of transfection, total protein was extracted and protein expression was analyzed by Western blotting. The *siRNA* to target *PRNP* and a scrambled sequence were synthesized by Bioneer (Bionear, Daejeon, Korea).

### 4.6. SOD Activity

MSCs were harvested from the culture dish by scraping with a rubber policeman on ice. The cells were extracted using extraction buffer. Cell lysates (50 µg) were allowed to react with superoxide dismutase, immediately after which signals were measured every minute by ELISA reader (BMG labtech, Ortenberg, Germany) at the optical density 450 nm for 10 min.

### 4.7. Catalase Activity

MSCs protein extracts (40 µg) were incubated with 20 mM H_2_O_2_ (*v*) in 0.1 M Tris-HCl (Sigma-Aldrich) for 30 min. Next, 50 mM Amplex Red reagent (Thermo Fisher Scientific) and 0.2 U/mL of horseradish peroxidase (Sigma-Aldrich) were added and incubated for 30 min at 37 °C. Changes in the absorbance values associated with H_2_O_2_ degradation were measured by ELISA reader (BMG Labtech, Ortenberg, Germany) at 563 nm.

### 4.8. Annexin V/PI Assay and Flow Cytometry-Based Analysis

Apoptosis in MSCs was assessed with a Cyflow Cube 8 (Partec, Munster, Germany) after staining the cells with Annexin V-FITC and propidium iodide (PI) (De Novo Software, Los Angeles, CA, USA). Data was analyzed using standard FSC Express (De Novo Software, Los Angeles, CA, USA).

### 4.9. Statistical Analysis

Results are expressed as mean ± standard error of the mean (SEM). All of the experiments were analyzed by one-way analysis of variance (ANOVA). Some comparisons of ≥3 groups were made using the Dunnett’s or Tukey’s post hoc test. A *p* value < 0.05 was considered statistically significant.
